# Automatic Classification Using Machine Learning for Non-Conventional Vessels on Inland Waters

**DOI:** 10.3390/s19143051

**Published:** 2019-07-10

**Authors:** Marta Wlodarczyk-Sielicka, Dawid Polap

**Affiliations:** 1 Institute of Geoinformatics, Department of Navigation, Maritime University of Szczecin, Waly Chrobrego 1-2, 70-500 Szczecin, Poland; 2 Marine Technology Ltd., ul. Roszczynialskiego 4/6, 81-521 Gdynia, Poland

**Keywords:** machine learning, image analysis, feature extraction, ship classification, marine systems

## Abstract

The prevalent methods for monitoring ships are based on automatic identification and radar systems. This applies mainly to large vessels. Additional sensors that are used include video cameras with different resolutions. Such systems feature cameras that capture images and software that analyze the selected video frames. The analysis involves the detection of a ship and the extraction of features to identify it. This article proposes a technique to detect and categorize ships through image processing methods that use convolutional neural networks. Tests to verify the proposed method were carried out on a database containing 200 images of four classes of ships. The advantages and disadvantages of implementing the proposed method are also discussed in light of the results. The system is designed to use multiple existing video streams to identify passing ships on inland waters, especially non-conventional vessels.

## 1. Introduction

Monitoring marine vessels is crucial to navigational safety, particularly in limited waters. At sea, vessel traffic services (VTS) are the systems responsible for supervision and traffic management [1]. In the case of inland waters, the support system used to track ships is called river information services (RIS) [2]. These systems use a variety of technologies to assist in observation, e.g., the automatic identification system (AIS), radars, and cameras [3]. The AIS is an automated tracking system that uses information concerning the position of the ship and additional data entered by the navigator, provided that the ship has an appropriate transmitter [4,5]. The international convention for the safety of life at sea (SOLAS convention) [6] developed by the International Maritime Organization requires that AIS transmitters be installed on vessels with a gross tonnage of 300 and more engaged in international voyages, vessels with a gross tonnage of 500 and more not engaged in international voyages, and passenger vessels. All other vessels navigating at sea and in inland waters cannot be unambiguously identified by traffic monitoring systems (for example, non-commercial craft, recreation craft, yachts, and other small boats).

No system or method is available at present for the automatic identification of small vessels. The only way to recognize them is to observe markings on their sides. RIS and VTS information systems have video monitoring, where cameras are mounted mainly on river bridges and in ports. It provides a useful opportunity to observe ships from different perspectives. However, the entire process is not automated. These systems are operated round the clock or during the day (in the case of daily navigation) by human workers. Therefore, to minimize cost, the automation of system operations is desirable. This research problem is a part of the SHREC project, the main purpose of which is to develop a prototype system for the automatic identification of vessels in limited areas [7,8]. At this stage, the authors propose a method to detect and classify vessels belonging to four classes based on an image database. The proposed method is a component of the system and forms part of the detection and recognition layer.

The recognition process is one of the most popular tasks due to the number of problems. Feature extraction is particularly important, thanks to which it is possible to further assign these features to a given object. However, quite often, two-dimensional images contain various kinds of additional elements such as sky, trees, or houses. In the case when an image is taken with a good camera, further objects can be blurred, which is a certain distortion causing a better extraction of the features of the closer elements. Unfortunately, this situation is not always available. Hence, scientists from around the world are modeling different solutions to get the best results. First of all, artificial intelligence techniques have developed quite dynamically in recent years. In [9], the authors presented the recent advances in convolutional neural networks, which are a popular tool for image classification. The authors noticed the growing popularity trend as well the modeling of more hybrids and modifications. In [10], the idea of using this mathematical model of neuron nets was used in medical diagnosis. Again in [11], the idea of long short-term memory was introduced as an improvement of the classic approach, which can be used for obtaining better efficiency. The problem of classification of water facilities was analyzed in [12,13], where deep representation for different vessels was examined. The problem of the amount of data in the classification process was analyzed in [14] by the use of data augmentation. Existing classification solutions apply to marine waters for large ships. It should be emphasized that, the designed system will be using multiple existing video streams to identify passing ships on inland waters, especially non-conventional vessels.

The remainder of this article is organized as follows: Section 2 presents the problem of detecting the features of ships in images using different filters, and Section 3 contains a description of the convolutional neural network used for classification. Section 4 details the proposed ship classification system model, and Section 5 describes our experiments and the conclusions of this study. The main goal of this paper is to propose a detection algorithm based on parallel image processing that allows the creation of two different samples from one image and the architecture of a deep convolutional neural network. Our tests are the basis during the construction of the SHREC system architecture. 

## 2. Detecting Features of Ships

The proposed technique of detecting features of ships in images is based on the parallel processing of a given image using different filters. This processing allows the distortion of images in such a way that only important elements giving features are mapped. Images obtained from the parallel filtering process are used to find features that appear in both images with different filters. The Hessian matrix was used for comparison and detection. The proposed technique relies on classical image processing to reduce the area used for further classification. The features of vessels were detected in order to minimize the amount of data in images such as background or surroundings. We used the idea of key points to detect only significant elements. An additional advantage of the proposed detection method is extension of the number of samples in each class. The proposed detection technique returns two rectangles, where both are interpreted as different images. Before being used in a subsequent classification process, they are scaled down in size, resulting in additional distortion.

The first of the images is blurred using a fast box algorithm [15]. To formulate the expression for this action, a convolution needs to be defined. The convolution of two functions is called an operation, the result of which is a new function *h*(·). Thus, image convolution consists of a moving window (matrix) with function values *g*(·) called a filter along function value *f*(·) that calculates the sum of products of the obtained values. This can be formally described as follows (in a discrete way):

(1) $$h\left[i,j\right]=\left(f\;*\;g\right)\left[i,j\right]=\overset{i+r}{\underset{y=i-r}{\sum }}\overset{j+r}{\underset{x=j-r}{\sum }}f\left[y,x\right]\;g\left[y,x\right]$$

The discreteness implies that the functions *f*(·) and *g*(·) are matrices. The first one stores the pixel values of the processed image, and the second one is much smaller (its dimensions are 3 × 3 or 4 × 4 and are marked as *r* × *r*). Its coefficients are constant and predetermined for the corresponding filter. For Gaussian blur, the following matrix is obtained:

(2) $$g=\left[\cdot ,\cdot \right]=\left(\begin{array}{ccc} \frac{1}{16} & \frac{1}{8} & \frac{1}{16}\\ \frac{1}{8} & \frac{1}{4} & \frac{1}{8}\\ \frac{1}{16} & \frac{1}{8} & \frac{1}{16} \end{array}\right)$$

Box blur involves blurring certain areas (the size of the filter) and allows for multiple instances of blurring in a given area (approximated) compared with the transition of the complete image several times. This solution reduces the number of calculations. The blur distorts the image, but to enhance the features, the image is converted into grayscale. This is done by replacing each pixel, *pxl*, (composed of the three components of the RGB model (Red–Green–Blue)) according to the following formula:

(3) $$R\left(pxl\right)=G\left(pxl\right)=B\left(pxl\right)=\frac{R\left(pxl\right)+G\left(pxl\right)+B\left(pxl\right)}{3}$$

Then, the contrast and brightness are increased. Changing the contrast allows for the darkening of the dark pixels and the brightening of the bright ones, and can be achieved using

(4) $$C\left(pxl\right)=\left[C\left(pxl\right)\;128\right]\cdot \eta +128$$

where *η* ≤ 0 is a threshold, and *C(pxl)* is a specific function defined as a component of the RGB model. The brightness of this image is increased, and all colors are approximated to white. The effect is the elimination of pixels close to white in color:

(5) $$C\;\left(pxl\right)=C\left(pxl\right)+\sigma$$

where *σ* is a threshold. The colors of the pixels are then inverted, which allows for better visualization of the other elements. To this end, the process involves converting each component of the pixel as

(6) $$C\left(pxl\right)=255-C\left(pxl\right)$$

It is easy to see that the inverted color highlights small elements that would otherwise be invisible. They can be removed using two operations: Improving the gamma value and binarization. Gamma correction with a coefficient *γ* allows for the scaling of the brightness of the image (that is, the other elements):

(7) $$C\left(pxl\right)=255\cdot \frac{C{\left(pxl\right)}^{\gamma }}{255}$$

The last step in processing the first image is its binarization, i.e., the conversion of all pixels into white or black. The conversion is carried out by calculating the average value of all components of a given pixel, *pxl*, and checking if it is within the range 〈0,α〉 or (α,255〉 as

(8) $$\left\{\begin{array}{c} \frac{\sum_{C\epsilon \left\{R,G,B\right\}}C\left(pxl\right)}{3}\leq \alpha \;then\;C\left(pxl\right)=0\\ \frac{\sum_{C\epsilon \left\{R,G,B\right\}}C\left(pxl\right)}{3}>\propto \;then\;C\left(pxl\right)=255 \end{array}\right.$$

The second image is processed in almost the same way using other coefficients, such as the threshold values. The most significant difference is the absence of binarization on the second image. Images created in this way can be used to detect features that can help locate the ship. For this purpose, the SURF algorithm (Speeded-Up Robust Features) is used [16]. In a given image, it searches for features by analyzing the neighborhood based on the determinant of the Hessian matrix defined as follows:

(9) $$H\left(p,\omega \right)=\left[\begin{array}{cc} L_{xx}\left(p,\omega \right) & L_{xy}\left(p,\omega \right)\\ L_{xy}\left(p,\omega \right) & L_{yy}\left(p,\omega \right) \end{array}\right]$$

where *L_ij_(p, ω)* represents the convolution of the integrated image *I* at point *p = (x, y)* with smoothing using the Gaussian kernel through parameter *ω*. The coefficients of this matrix can be represented by the following formulae:

(10) $$L_{xx}\left(p,\omega \right)=I\left(p\right)\frac{\partial^{2}}{\partial x^{2}}g\left(\omega \right)$$

(11) $$L_{yy}\left(p,\omega \right)=I\left(p\right)\frac{\partial^{2}}{\partial y^{2}}g\left(\omega \right)$$

(12) $$L_{xy}\left(p,\omega \right)=I\left(p\right)\frac{\partial^{2}}{\partial xy}g\left(\omega \right)$$

The aforementioned integrated image is an indirect representation called rectangle features. For a given position *p = (x, y)*, the sum of all pixels in the original image *I’(*·) is calculated as

(13) $$I\left(p\right)=I\left(\left(x,y\right)\right)=\overset{i\leq x}{\underset{i=0}{\sum }}\overset{j\leq y}{\underset{j=0}{\sum }}I^{\prime }\left(i,j\right)$$

The algorithm chooses points that form the trace of the matrix shown in Equation (9) and the local extreme. For this purpose, the values of the Hessian determinant and the trace are determined:

(14) $$det\left(H\left(p,\omega \right)\right)=\omega^{2}\left(L_{xx}\left(p\right)L_{yy}\left(p\right)-L_{xy}^{2}\left(p\right)\right)$$

(15) $$trace\left(H\left(p,\omega \right)\right)=\omega \left(L_{xx}\left(p\right)+\;L_{yy}\left(p\right)\right)$$

In this way, the key points are determined, i.e., the second image is processed without binarization. Then, for each key point found, its color is checked on the second image (after binarization). As a result, two sets of points are obtained belonging to one of two colors—black or white. However, the number of points can be very large, and therefore it is worth minimizing them using the average distance for the entire set. 

It is assumed that the set of key points for one of the colors is marked as $\left\{p^{\left(0\right)},p^{\left(1\right)},\;\ldots ,\;p^{\left(n-1\right)}\right\}$. The average distance ξ for all points in the set is calculated using the Euclidean metric defined as

(16) $$\xi =\frac{\sum_{i=0}^{n-1}\sum_{j=0}^{n-1}\sqrt{{\left(p_{x}^{\left(i\right)}-p_{x}^{\left(j\right)}\right)}^{2}+{\left(p_{y}^{\left(i\right)}-p_{y}^{\left(j\right)}\right)}^{2}\;}}{{\left(n-1\right)}^{2}-\left(n-1\right)}$$

The calculated distance is used to analyze all points in the set. If, for any point, there is a neighbor at a distance smaller than ξ, the given point remains in the set. Otherwise, it is removed.

**Figure 1:**
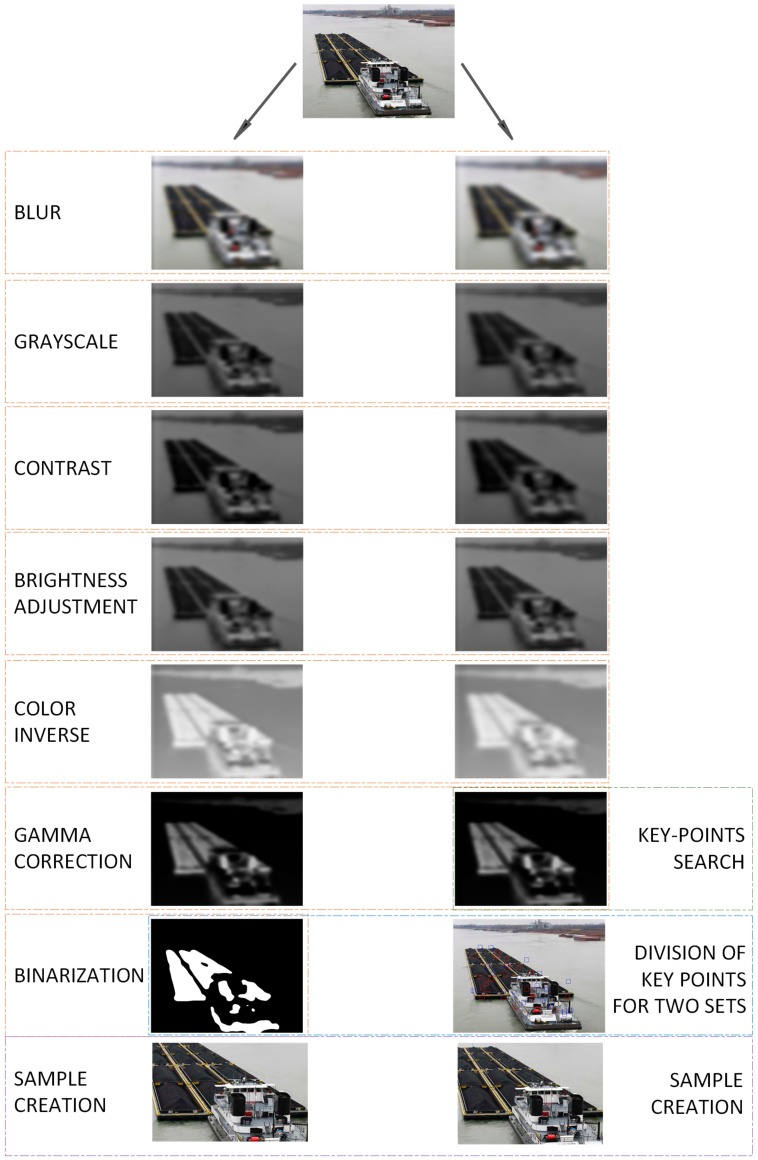
Graphical hierarchy of image processing.

Finally, the method returns two sets of points that can be called features of the processed image. These points are used to find the maximum number of dimensions of the found object by finding the largest and smallest values of the coordinates *x* and *y*. The image between these extremes is cut out and used as a sample in the ship classification process. The model of image processing is shown in Figure 1.

## 3. Convolutional Neural Network for Classification

The image in computing is a tensor, i.e., a mathematical object described by three components *w × h × d*. The first two components form the dimensions of the image, i.e., width and height, and the third is its depth. In the classic approach to this subject, the depth of the image is the number of components. Thus, for the RGB model, there are three values and the depth is three. As a result, three matrices are created in which the numerical values of a given color are stored. Such data can be used in the recognition process using artificial neural networks called convolutional neural networks. Such structures are inspired by the action and mechanisms of the cerebral cortex [10,17].

This input structure accepts a graphic file and processes it to classify it. It is composed of several types of layers, where the first is called the convolutional layer. It is interpreted as a filter of a given size *k × k*. This filter moves on the image and modifies it. However, it is worth noting that by dividing the image according to filters of different sizes, a grid of rectangles can be obtained. Each rectangle represents a part of the image to which a weight w is assigned, that is, a numerical value used for further training. Each part is interpreted as a neuron and returns the following value:

(17) $$f\left(\overset{k}{\underset{i=0}{\sum }}\overset{k}{\underset{j=0}{\sum }}w_{i,j}\cdot a_{x+i,y+j})\right)$$

where *f* (∙) is the activation function, and *a_x,y_* is the activation value for the output from position *(x, y)*.

The convolutional layer is responsible for the extraction of features and eliminating redundancy in the data. It is easy to see that the grids can overlap, which may result in multiple appearances of the same fragment. To prevent this, a pooling layer is used to reduce the size of the image by extracting only the most appropriate features. For this selection, the maximum value is typically used. Both layers may occur several times depending on the problem and its size (understood as image size). A third type of layer, a fully connected layer, is also used. It is a classical neural network, the input to which consists of data from the previous layer (for each pixel, there are three values). The operation of the classical neural network can be described as the processing of numerical values. Neurons are arranged in columns called layers, where neurons between neighboring layers are related to one another by weighted connections. For each neuron, the output value *x_i_* and weight *w* from the previous neurons are sent. The value of each neuron is calculated as in Equation (17), that is, as the sum of the products of weights and values of neurons from the previous layer rescaled by the activation function. 

As the output of the entire network, values are obtained that are not yet a probability distribution. They are normalized using the softmax function. The approximate values should indicate the assignment to the class predicted by the classifier.

However, all weights in the neural network are assigned in a random manner, which causes the classifier to return meaningless information. To remedy this, an important step is to train the classifier. The training process consists of checking the assignment returned by the network and comparing it with the correct one. The comparison is made using a loss function. The idea of training is to minimize the loss function by modifying weights in the network. An example of an optimization algorithm used for training is adaptive moment estimation, often called the Adam optimizer [18]. The algorithm is based on calculating the variance *v* and mean *m* for each iteration *t*. If a single weight is *w^(t)^,* the values have the following form:

(18) $$m_{t}=\beta_{1}m_{t-1}+\left(1-\beta_{1}\right)g_{t}$$

(19) $$v_{t}=\beta_{2}v_{t-1}+\left(1-\beta_{2}\right)g_{t}^{2}$$

where $\beta_{1}\;\mathrm{and}\;\beta_{2}$ are distribution values. Using them, the following correlations can be defined:

(20) $${\hat{m}}_{t}=\frac{m_{t}}{1-\beta_{1}^{t}}$$

(21) $${\hat{v}}_{t}=\frac{v_{t}}{1-\beta_{2}^{t}}$$

The above correlation formulae are used to update weights in the network using the following equation:

(22) $$\theta_{t+1}=\theta_{t}-\frac{\eta }{\sqrt{{\hat{v}}_{t}}+\epsilon }{\hat{m}}_{t}$$

where *η* is the training rate, and ϵ is a very small value preventing an indeterminate form.

## 4. Model of Ship Classification System

The idea of the proposed system is to create software that can process real-time video samples and categorize ships appearing in them. The camera recording the image can be placed in any position, such as a water buoy or a bridge. The video being recorded is composed of many frames, which may not necessarily feature a vessel. Moreover, it makes no sense to process each frame—one second of a recorded video usually contains 24 frames, it is not possible to process this amount of data in real time. 

Hence, image processing can be minimized to one frame every one or two seconds. This prevents the task queue from overloading and blocking. However, keeping the camera in one position leads it to record the same elements in space. Therefore, to avoid searching for ships in nearly identical images, a pattern was created to avoid the need for continuous processing. The idea of this solution is to find key points at different times of the day and in different weather (for this purpose, the SURF algorithm was used). If a given frame has only points that coincide with the previous pattern, the frames do not need to be analyzed in terms of searching for a floating vehicle. If there are greater deviations from the standard (for example, a greater number of points), image processing should be performed as described in Section 2, and classification should then be performed using the convolutional neural network described in Section 3.

The proposed data processing technique allows reducing the area of the incoming image. An additional advantage is the creation of two images, which are created by parallel image processing. In the classification process, the images are reduced in order to normalize the size of all samples. The effect of this action is to obtain two images that are distorted by size reduction. 

## 5. Experiments

The previous sections described the image processing model to extract only fragments and the model of the classifier that categorizes incoming images into selected classes. Depending on the number of classes, this is the output of the network. For *n* classes, there are *n* neurons in the last layer, and the classes are saved as *n*-element vectors consisting of *n* − 1 zeros and a single one. For each class, the “1” is in a different position in the vector. The experimental part is divided into two parts: Tests on public images and tests on real data collected by means of cameras mounted on bridges and placed on the bank in the port of Szczecin. 

### 5.1. Experiments on Test Data

To check the operation of the proposed method, the authors used an image database divided into four classes, each of them composed of 200 samples. The classes represented four types of ships—a boat, a motor yacht, a navy ship, and a historic ship. The image processing method presented in Section 2 was tested on different values of parameters in the range {0, 15}. The adjustment of these values for the system led to the setting of an average number of key points. All coefficients were used in different combinations, and the authors selected one that returned approximately 15–20 key points (after processing). The selected configuration for image processing is presented in Table 1. This number is not accidental because the authors noticed that if the number of key points was small, the worse area was returned. On the contrary, when the number of key points was large, the original image was returned. This is why the configuration was chosen in an empirical manner.

**Figure 2:**
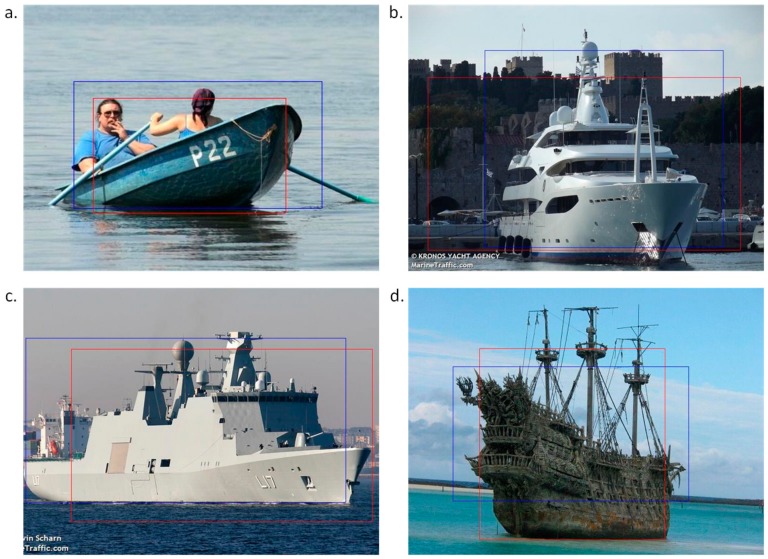
Sample examples of images, where the rectangles indicate the extracted images: (**a**) boat; (**b**) motor yacht; (**c**) navy ship; and (**d**) historic ship.

In this way, the best configuration was selected and the files were processed. It is worth noting that two images were always generated from one sample, as presented in Figure 2. Before these samples were used for training, images among them that did not contain vessels were removed. The database of each class (composed of 200 samples) was thus extended to an average value of 320 images (first class, 343; second class, 316; third class, 324; and fourth class, 299). The obtained samples did not always contain the entire vessel, but only fragments or other objects in the image. This was important for the training process because it could not be guaranteed that an incoming vessel would be oriented in a particular manner. In addition, using the same method of extraction yielded a similar sample.

The proposed technique for processing and extracting features allowed for an average of 320 samples, whereas the ideal solution would return exactly 400 (double the amount from the entry database). However, the different conditions in which the images were taken (in terms of quality, distance, and exposure) and the background posed problems. For ships in high seas, the extraction was perfect. However, a large part of these samples was set against the background of forests or cities. Thus, the recorded average extraction efficiency of 80% is a good result. It might have been affected by incorrect filter configuration. An empirical approach to the evaluation of the obtained data indicated the best adjustment of these values.

**Figure 3:**
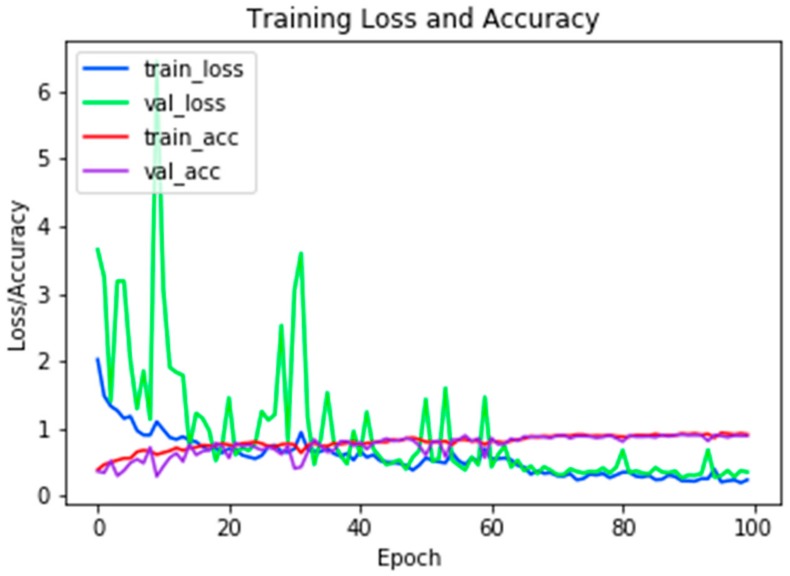
Learning history of the classifier obtained using the created database.

Table 2 presents the structure of the classification network. The layers were selected in order to obtain the highest effectiveness. It was noted that the proposed technique for the division of samples in the ratio 80:20 (training:verification) and the learning rate of *η* = 0.001 gives the best results. We trained the classifier during 100 epochs. The resulting efficiency was 89%. A graph of the training is shown in Figure 3. 

**Figure 4:**
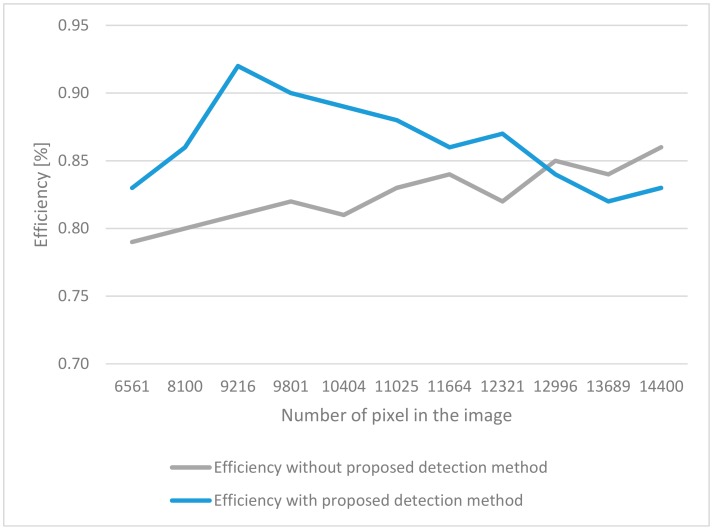
Measurement of the effectiveness of the techniques relative to the size of the incoming image.

To more accurately verify the operation of the classifier, 118 false samples were added to the database. In our experiments, different sizes of images were analyzed. For a given architecture, we tested the classifier in order to achieve the best possible accuracy. The obtained measurements are presented in Figure 4. 

**Figure 5:**
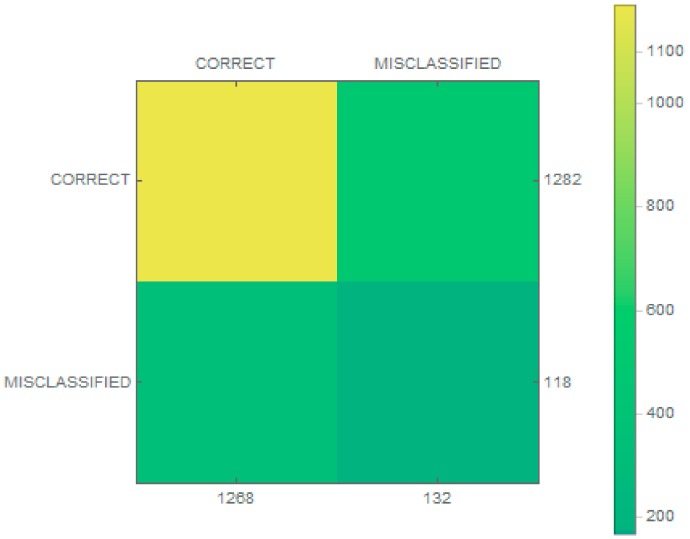
The confusion matrix of the classification model.

**Figure 6:**
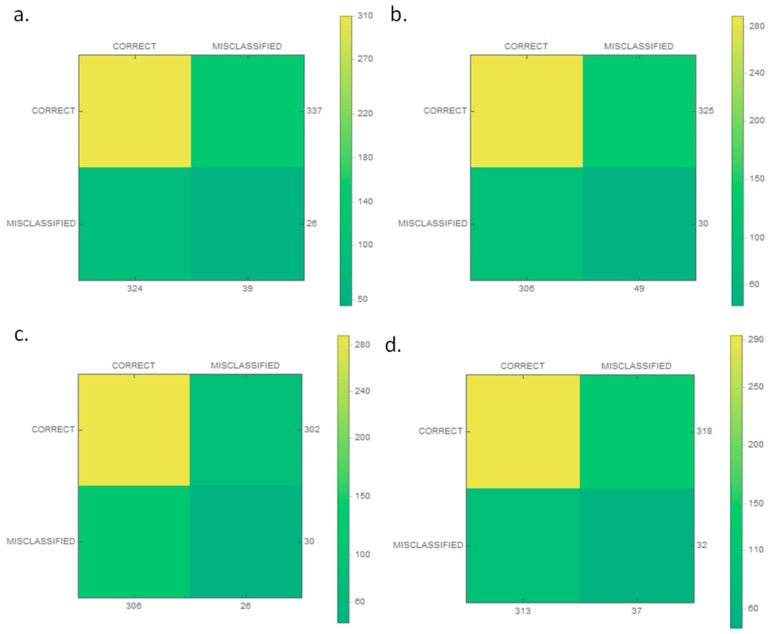
The confusion matrices of the classification model for each class: (**a**) boat; (**b**) motor yacht; (**c**) navy ship; and (**d**) historic ship.

The classification results were then checked and are presented as a confusion matrix in Figure 5. In addition, matrices for individual classes were made, as shown in Figure 6. The proposed classifier categorized every wrong sample into one of the classes of vessels. A condition was thus added (to the extraction stage) that if no key point was found in an image, the image was rejected.

Based on the results, the authors determined the accuracy, overlap, sensitivity, specificity, and the F-measure. The results are presented in Table 3. 

The resulting effectiveness was nearly 92%, which is high considering that a small number of samples were used for training. A higher value was obtained for sensitivity, which implies accurate prediction of true samples. The F1 score was also high, which indicates that the classifier was efficient. Considerably worse results were obtained for specificity, nearly 0.541, implying that the classification of true negatives was substandard. The same result was obtained for the rate of false omission, indicating that a large portion (almost 30%) of the false samples had been rejected.

**Figure 7:**
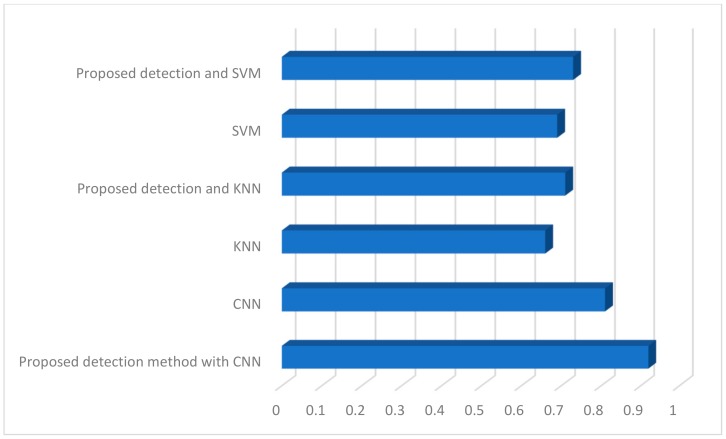
Comparison of different classification techniques on a selected database. SVM—support vector machine; KNN—k-nearest neighbors; CNN— convolutional neural network.

As part of the conducted research, classification tests on a given database were performed with and without the proposed detection method and the results are presented in Figure 7.

First of all, we made a test that uses a convolutional neural network without detection. The efficiency was about 80%. Again, for classic classifiers such as k-nearest neighbors (KNN) and support vector machine (SVM), the results were similar. In both cases, the proposed detection technique increased the value of effectiveness. However, the highest results were obtained for the proposed technique along with a convolutional neural network.

### 5.2. Experiments on Real Data 

**Figure 8:**
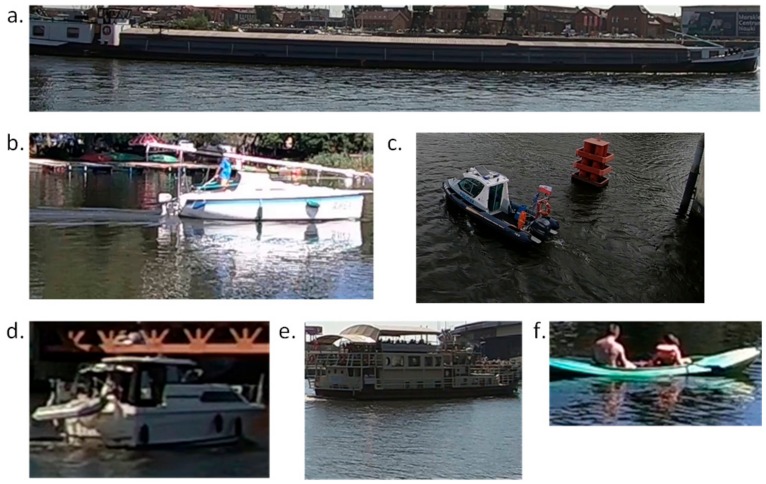
Sample examples of images: (**a**) barge; (**b**) yacht; (**c**) marine service; (**d**) motorboat; (**e**) passenger; and (**f**) other.

In the next step, we analyzed the proposed solution on real data. To test the proposed solution in real conditions, we manage to create a database contained 18,361 image files that stored six classes of boats—barge (1703 images), yacht (1489 images), marine service (2362 images), motorboat (6980 images), passenger (3629 images), and other kinds (2198 images). The data were obtained by the setting of cameras mounted on bridges and placed on the bank in Szczecin in Poland. The resulting video data were processed with detection to get video frames containing the passing ship on inland waters. Within one second, a maximum of one frame was collected. For the needs of the experiments, a maximum of 20 photos of one ship crossing the bridge were left in the database. The examples of this images are presented in Figure 8.

**Figure 9:**
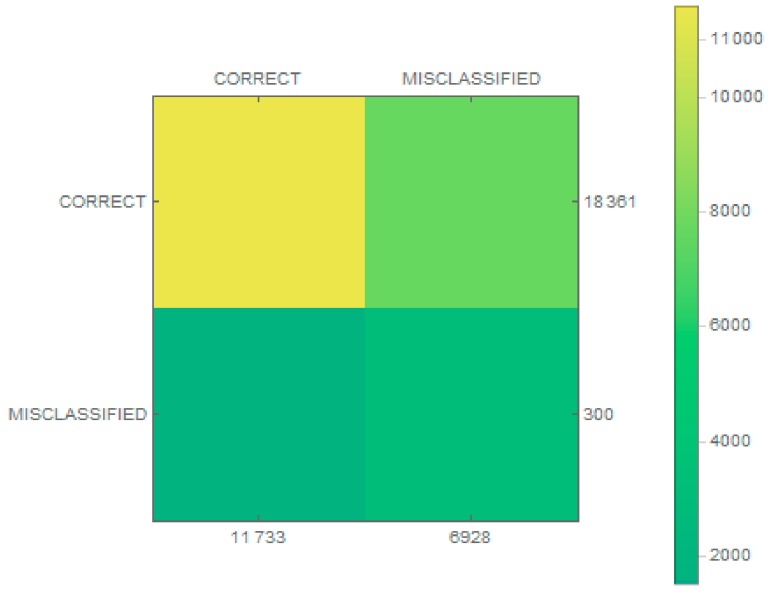
The confusion matrix of the classification model for real data.

**Figure 10:**
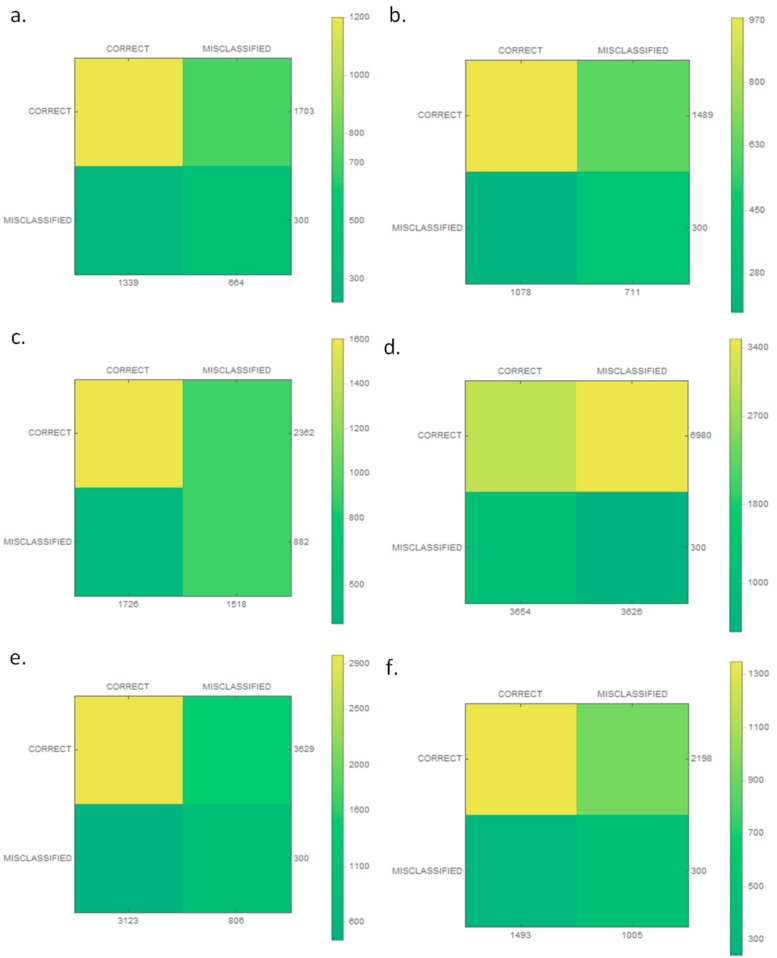
The confusion matrices of the classification model for each class: (**a**) barge; (**b**) yacht; (**c**) marine service; (**d**) motorboat; (**e**) passenger; and (**f**) other.

Images obtained at the stage of detection differ from each other in size and quality. As part of the research, the same classification architecture was used, which is presented in Table 2, with one difference being that there were six neurons in the output layer because of the number of classes. For the purposes of conducting experiments, the images within each class were divided in the ratio 80:20 (training:verification), with a training coefficient\eta = 0.001 and 100 iterations. After the training process, the database was enlarged by an additional 300 pictures that did not fit into any of the six classes. For each of the classes, plus the previously mentioned fake images, the efficiency of the trained classifier was checked. The results in the form of confusion matrices are presented in Figure 9 for the classifier and for each class in Figure 10.

Statistical tests for each class are presented in Table 4. In the case of average performance of the proposed classification, accuracy reached 63%. As in the case of test data, the higher value was obtained for sensitivity. In addition, the recall is on the same level. The F1 score was also high, which indicates that the classifier is efficient. Here, we also see that worse results were obtained for specificity and miss rate.

**Figure 11:**
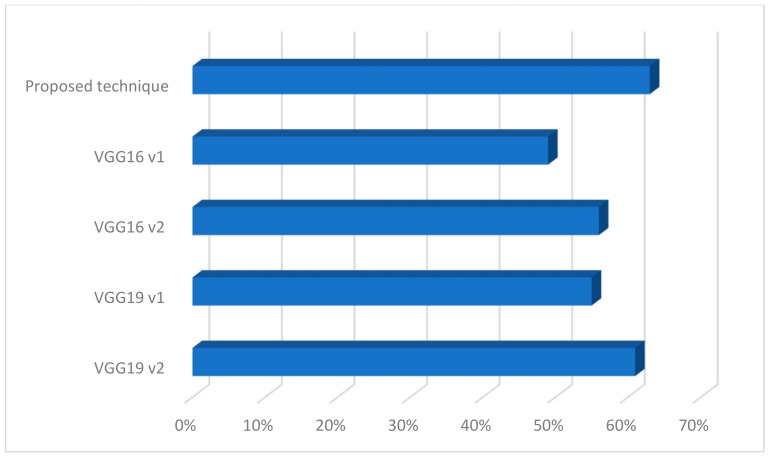
Comparison of different convolutional neural networks on a selected database.

Next, for the database thus created, the operation of the proposed method was verified with other classic architectures of the convolution network as shown in Figure 11.

In our experiments, we used a strategy to train some layers and leave others frozen in existing models of CNN such as VGG16 and VGG19 [19]. Such a strategy was used to transfer knowledge from previously trained classifiers so as not to train classifiers from scratch. While choosing to leave specific layers, we paid attention to possible overfitting. We have chosen a few examples of architectures to illustrate the proposed solution with existing ones. In the case of the first version (on the chart v1), the last five layers were frozen, and in the second case (on the charts v2), it was the last six layers. In both cases, two layers were added to the architecture as fully connected ones—in both cases, a first layer was composed of 512 neurons and a ReLu function, and the last one had six neurons. This architecture yielded less effectiveness than the proposed technique. Summarizing, the proposed solution can be used in the system being built. Although the classes are similar, the network was able to classify the images. However, the number of samples in the database should be increased.

## 6. Conclusions

The identification and categorization of ships in images can be very useful for creating a register of passing ships, observing boundaries, and even recording information on a catastrophic recorder. This paper proposed a model to extract features to identify marine vessels from a single frame of an image and a deep learning-based method to categorize them. The authors analyzed only four categories of vessels, each of which was initially composed of 200 sample images. Features of ships were detected in images through the parallel processing of an image using different filters. The aim was to distort images so that only important elements yielded feature maps. Both images obtained from the parallel filtering process were used to find features common to them using different filters. The Hessian matrix was used for this. For feature extraction, the database was extended to include additional samples used to train the classifier. During classification, an image was assumed to be a tensor with its number of components as its depth (RGB model). The convolutional neural network was used for the recognition process. Its structure was inspired by the action and mechanisms of the cerebral cortex. Following tests and the reconfiguration of the network, the obtained efficiency for test data was nearly 90%, which is a very good result considering the small number of samples. In the case of a real database with six classes the average accuracy reached 63%. The proposed solution will be implemented in the identification system being built. However, the database should be increased.

In the SHREC system, the camera stream is analyzed using image processing methods for detection, classification, and text recognition to properly identify a vessel. The method for ship classification presented in this article will form part of ongoing research in the SHREC project. Future work will focus on improving the proposed classification method. The authors intend to modify the parameters of the method to be able to classify more types of ships. For this purpose, a more extensive database of images of ships is being gathered using video cameras at varying resolutions. 

## Figures and Tables

**Table 1:** The selected configuration for image processing.

Filter	First Image	Second Image
Blur	8	8
Grayscale	yes	yes
Contrast	5	5
Brightness	15	15
Inverse	yes	yes
Gamma	6	7
Binarization	yes	no

**Table 2:** Architecture of convolutional neural network.

Type of Layer	Shape
Convolutional 3 × 3	(None, 96, 96, 32)
Activation—ReLU function	(None, 96, 96, 32)
Batch normalization	(None, 96, 96, 32)
Max pooling 3 × 3	(None, 32, 32, 32)
Dropout 0.25	(None, 32, 32, 32)
Convolutional 3 × 3	(None, 32, 32, 64)
Activation—ReLU function	(None, 32, 32, 64)
Batch normalization	(None, 32, 32, 64)
Convolutional 3 × 3	(None, 32, 32, 64)
Activation—ReLU function	(None, 32, 32, 64)
Batch normalization	(None, 32, 32, 64)
Max pooling 2 × 2	(None, 16, 16, 64)
Dropout 0.25	(None, 16, 16, 64)
Convolutional 3 × 3	(None, 16, 16, 128)
Activation–ReLU function	(None, 16, 16, 128)
Batch normalization	(None, 16, 16, 128)
Convolutional 3 × 3	(None, 16, 16, 128)
Activation—ReLU function	(None, 16, 16, 128)
Batch normalization	(None, 16, 16, 128)
Max pooling 2 × 2	(None, 8, 8, 128)
Dropout 0.25	(None, 8, 8, 128)
Flatten	(None, 8192)
Dense 1024	(None, 1024)
Activation—ReLU function	(None, 1024)
Batch normalization	(None, 1024)
Dropout 0.5	(None, 1024)
Dense 4	(None, 4)
Activation—Softmax function	(None, 4)

**Table 3:** Mean quality measures for the trained classifier.

Measure	Boat	Motor Yacht	Navy Ship	Historic Ship	Mean
Accuracy	0.915	0.909	0.928	0.91	0.916
Sensitivity	0.957	0.958	0.962	0.96	0.959
Specificity	0.679	0.558	0.659	0.438	0.583
Precision	0.944	0.939	0.957	0.941	0.945
Negative prediction rate	0.735	0.649	0.692	0.538	0.654
Miss rate	0.043	0.042	0.037	0.04	0.041
False discovery rate	0.056	0.061	0.043	0.059	0.055
False omission rate	0.265	0.351	0.308	0.462	0.346
F1 score Recall	0.951 0.944	0.948 0.939	0.96 0.957	0.95 0.941	0.952 0.945

**Table 4:** Mean quality measures for the trained classifier.

Measure	Barge	Yacht	Marine Services	Motorboat	Passenger	Other
Accuracy	0.668	0.603	0.648	0.502	0.795	0.598
Sensitivity	0.882	0.83	0.901	0.963	0.95	0.897
Specificity	0.217	0.164	0.139	0.046	0.18	0.146
Precision	0.705	0.657	0.679	0.5	0.821	0.613
Negative prediction rate	0.463	0.333	0.41	0.557	0.473	0.483
Miss rate	0.118	0.17	0.099	0.037	0.05	0.103
False discovery rate	0.295	0.343	0.321	0.5	0.179	0.387
False omission rate	0.537	0.667	0.59	0.443	0.527	0.517
F1 score Recall	0.783 0.882	0.733 0.83	0.774 0.901	0.658 0.963	0.881 0.95	0.728 0.897
